# An Efficient Algorithm for Maximizing Range Sum Queries in a Road Network

**DOI:** 10.1155/2014/541602

**Published:** 2014-07-24

**Authors:** Tien-Khoi Phan, HaRim Jung, Ung-Mo Kim

**Affiliations:** ^1^School of Information and Communication Engineering, Sungkyunkwan University, 2066 Seobu-ro, Jangan-gu, Suwon 440-746, Republic of Korea; ^2^Department of Computer Engineering, Sungkyunkwan University, Republic of Korea

## Abstract

Given a set of positive-weighted points and a query rectangle *r* (specified by a client) of given extents, the goal of a maximizing range sum (MaxRS) query is to find the optimal location of *r* such that the total weights of all the points covered by *r* are maximized. All existing methods for processing MaxRS queries assume the Euclidean distance metric. In many location-based applications, however, the motion of a client may be constrained by an underlying (spatial) road network; that is, the client cannot move freely in space. This paper addresses the problem of processing MaxRS queries in a road network. We propose the external-memory algorithm that is suited for a large road network database. In addition, in contrast to the existing methods, which retrieve only one optimal location, our proposed algorithm retrieves all the possible optimal locations. Through simulations, we evaluate the performance of the proposed algorithm.

## 1. Introduction

With the widespread use of mobile computing devices [1–7], location-based services [8] have attracted much attention as one of the most promising applications whose main functionality is to process location-related queries on spatial databases. Most traditional research in spatial databases have focused on finding nearby data objects (e.g., range queries, nearest neighbor queries [9], etc.), rather than finding the best location to optimize a certain objective. Recently, a maximizing range sum (MaxRS) query was introduced in [10]. This query is useful in many location-based applications such as finding the most representative place in a city with a limited reachable range for a tourist or finding the best location for a pizza store with a limited delivery range. Given a set of positive-weighted points and a query rectangle  *r* (specified by a client) of a given size, the goal of a MaxRS query is to find the optimal location of  *r* such that the sum of the weights of all the points covered by  *r* is maximized.

**Figure 1:**
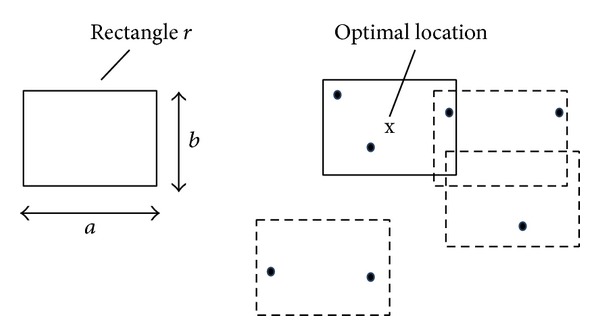
Example of the MaxRS query.

Figure 1 shows an example of the MaxRS query, where the size of the query rectangle  *r* is *a* × *b* and all the points are assumed to have the same weight and be equal to 1. In the figure, the center of the solid-lined rectangle is the optimal location of  *r* because the solid-lined rectangle covers the largest number of points (i.e., 3).

**Figure 2:**
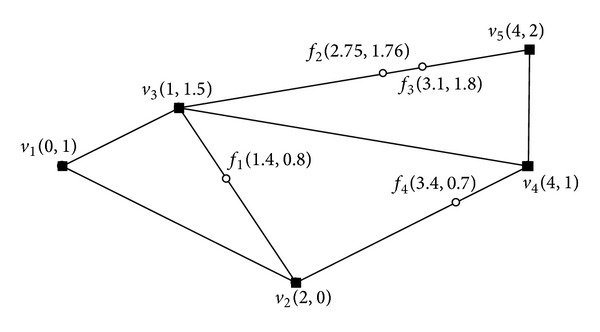
An example of road network.

To process MaxRS queries, Choi et al. [10] proposed an external-memory algorithm, while Imai and Asano [11] an internal-memory algorithm. Tao et al. [12] proposed the solution for approximate MaxRS queries, each of which retrieves a rectangle *r* whose covered-weight is at least (1 − *ε*)m*, where m* is the optimal covered-weight and *ε* is an arbitrary constant between 0 and 1. All of these studies aim at Euclidean spaces. In many real-life location-based services, however, the motion of a client may be constrained by an underlying (spatial) road network; that is, the client cannot move freely in space. Consider the scenario of a tourist service as an example, where a tourist (i.e., client) tries to find the hotel whose location is close to as many sightseeing spots as possible (e.g., maximum is 1.5 km walking from the hotel). In this scenario, a MaxRS query can be applied. However, the existing MaxRS query processing methods cannot be applied in this scenario because the distance between the hotel and each sightseeing spot is confined by the underlying (spatial) road network, and thus the actual distance between two locations can differ significantly from their Euclidean distance. We can see this significant difference in Figure 2, where the Euclidean distance between *f*_2_ and *f*_4_ is about 1.24, while for moving from *f*_2_ to *f*_4_ in real-life, we must pass through *v*_5_ and *v*_4_ with total length around 3.74, which is three times farther than Euclidean distance. With this problem in mind, we study, for the first time to the best of our knowledge, the problem of processing MaxRS queries in a road network, where the distance between two points is determined by the length of the shortest path connecting them (i.e., network distance [13]).

Figure 2 shows an example of the road network, which consists of 5 nodes (square vertices) and 7 edges. In the figure, there are 4 facilities (weighted points), each of which, denoted by  *f*, is associated with a positive weight  *w*(*f*) indicating the importance of  *f*. The numbers that appear in parenthesis next to nodes and facilities show their respective coordinates. Note that it is assumed in this paper that all the facilities must be located on edges of the road network. Then, a MaxRS query in a road network is defined as follows. Given a set of facilities and a radius  *r*, the MaxRS query finds all the locations  *p* (on a road network), which maximizes the total weights of all the facilities whose network distance to  *p* is less than or equals  *r*.

**Figure 3:**
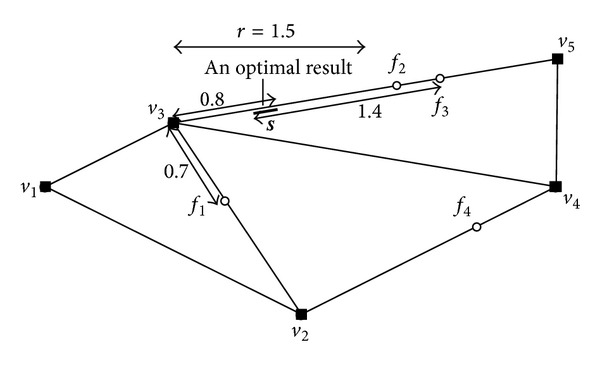
MaxRS query in road network.

In the case of road network in Figure 2, we have an example of MaxRS query with the radius 1.5 (km) in Figure 3 (the weight of each facility is 1). The distance between each point in the stage  *s* to three facilities *f*_1_, *f*_2_, and *f*_3_ is less than or equal to 1.5. And the total weight of all the facilities whose network distance to all points of stage  *s* is less than or equals 1.5 is 3, which is maximum in this scenario. Then, stage  *s* is an optimal result in this MaxRS query and user can choose any hotel on this stage.

In this paper, we propose the external-memory algorithm for MaxRS queries in a road network. The proposed algorithm is suitable for a large road network database. In addition, in contrast to the existing methods, which find only one optimal location, our proposed algorithm finds all the possible optimal locations. This can help clients of diverse interests choose their own best locations by considering other additional conditions.

The remainder of this paper is organized as follows. In Section 2, the problem is formally defined, and in Section 3, the details of the proposed algorithm are provided. In Section 4, the performance evaluation results are presented. In Section 5, some related work is reviewed. Finally, Section 6 concludes the paper.

## 2. Problem Formulation 

A road network is represented by an undirected graph *G* = (*V*, *E*), where *V* is a set of vertices (i.e., nodes) and *E* is a set of edges. Let *F* be a set of facilities, each of which, denoted by  *f*, is located on an edge (in *E*) and is associated with a positive weight  *w*(*f*).

**Definition 1 (network range and network radius).**

Network range  *p*(*r*) of a point  *p* in a road network consists of all points (in the network) whose network distance to  *p* is less than or equals the value  *r*, where  *r* is called the network radius of  *p*.

**Definition 2 (a MaxRS query in road network).**

Given  *G*, a set of positive-weighted points  *F*, and a network radius value  *r*, let  *p*(*r*) be the network range of a point  *p* in the network and  *F*_*p*(*r*)_ the set of facilities covered by  *p*(*r*). Then, a Maximizing range sum (MaxRS) query in a road network finds all points  *p* (in  *G*) that maximizes

(1) $$\begin{array}{c} \underset{f\in F_{p(r)}}{\sum }w\left(f\right). \end{array}$$

## 3. The Proposed Method

### 3.1. Preliminaries

In this subsection, we review the idea of transforming the max-enclosing rectangle query into the rectangle intersection query discussed in [14], which is the fundamental idea for processing MaxRS queries in Euclidean space [10].

**Definition 3 (max-enclosing rectangle query).**

Given a set of points  *O*, a rectangle  *r* with a given size, a max-enclosing rectangle query finds the location of  *r* such that  *r* encloses the maximum number of points in  *O*.

The MaxRS query calculates the total weight of points, while the max-enclosing rectangle query counts the number of points in rectangle. Note that when assuming all points have the weight being equal to 1, the result of the MaxRS query equals that of the max-enclosing rectangle query.

**Definition 4 (rectangle intersection query).**

Given a set of rectangles  *R*, a rectangle intersection query finds the area, where most rectangles overlap.

**Figure 4:**
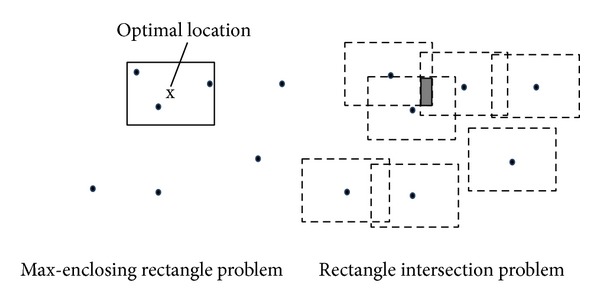
Example of transformation.

Figure 4 shows two examples of the max-enclosing rectangle query and the rectangle intersection query. It can be observed from the figure that the optimal location in the max-enclosing rectangle query can be any point in the most overlapped area (i.e., the gray area, where 3 rectangles overlap), which is the outcome of the rectangle intersection query.

**Figure 5:**
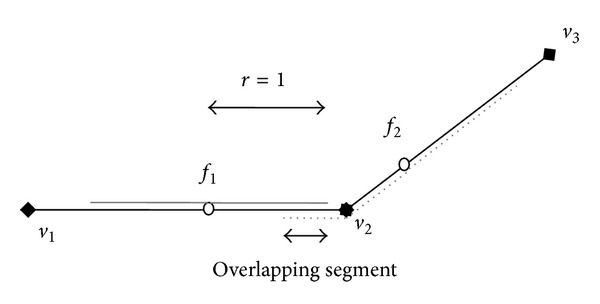
Max-segment in MaxRS query.

Our solution is based on the above idea. Consider an example of a MaxRS query in a road network shown in Figure 5. To simplify our discussion, we use a simple road network that consists of two edges (i.e., 〈*v*_1_, *v*_2_〉 and 〈*v*_2_, *v*_3_〉) and two facilities (i.e., *f*_1_ and *f*_2_) on two edges.

In this example, we assume that the weight of each facility is 1 and the network radius  *r* is 1. The gray solid segments in Figure 5 indicate the network range *f*_1_(*r*) of the facility *f*_1_, and gray dotted segments indicate the network range *f*_2_(*r*) of facility *f*_2_. Let  *S* be the set of all segments presented in the network range of all facilities in the road network. Then, we define the following two important notions for the MaxRS query in the road network.

**Definition 5 (location-weight).**

Let  *p* be the location in road network. The location-weight of  *p* with regard to  *S* equals the total weights of all the segments (in  *S*) that cover  *p*.

**Definition 6 (max-segment).**

The max-segment  *M* with regard to  *S* is a segment such that every point in  *M* has the same location-weight  *W*, and no point in the network has a location-weight higher than  *W*.

From the idea of the transformation mentioned before, we can see that the overlapping segment in Figure 5 is a max-segment. Because all max-segments in the network contain all the optimal locations (i.e., the result of the MaxRS query in the road network), we need to find all max-segments in the network to evaluate the MaxRS query.

### 3.2. Storage System

Similar to the disk-based storage model proposed in [13], the road network and the facility set are stored in a secondary storage.

**Figure 6:**
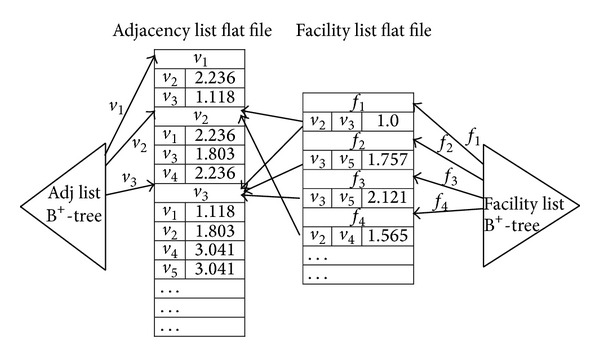
Disk-based storage model.

Figure 6 shows the files and indexes for the network and facility set. In this storage model, the network (adjacency list) is stored in a flat file, which is indexed by the B^+^-tree. For each node *v* (e.g., *v*_1_), besides the information of *v* (i.e., node identifier, coordinates), we also store the additional information of all adjacent nodes including adjacent node identifier and Euclidean distance between *v* and its adjacent node (e.g., length of edge 〈*v*_1_, *v*_2_〉 is 2.236). Similarly, the facility list is also stored in a flat file and indexed by the B^+^-tree. To support the algorithm efficiently, besides the information of each facility *f* (i.e., facility identifier, coordinates, and weight of facility), we store the additional information of the edge that contains *f* including start node identifier, end node identifier, and the Euclidean distance (offset) between start node and *f* (e.g., start node of *f*_1_ is *v*_2_, end node of *f*_1_ is *v*_3_, and length of segment 〈*v*_2_, *f*_1_〉 is 1.0).

### 3.3. Main Algorithm

#### 3.3.1. Overview

Our algorithm is based on the idea mentioned in Section 3.1. From each facility *f*, we generate segments that cover the network range *f*(*r*). The segments generated by facility *f* will have the weight of *f*, namely, *w*(*f*). These segments are organized in a seg-file. Then, we process the seg-file to find out all max-segment. The following three main steps constitute the proposed algorithm:

1. generating segments;
2. inserting segments into seg-file;
3. processing seg-file to find max-segments.

#### 3.3.2. Generating Segments

In this step, we generate segments from all facilities of facility flat file. For each facility *f*, we generate the segments, which cover the overall network range *f*(*r*). This process is described in Algorithm 1. First of all, we retrieve the information of the edge that contains *f*, start node, and end node. Then, we generate the segments at the start node side first (lines 8–16), after which we generate the segments at the end node side (lines 17–26). If the distance between *f* and the start node is greater or equals the network radius *r*, we only need to generate one segment with the length being equal to *r* (lines 9-10). On the contrary, we generate the segment between *f* and the start node (the length is equal to the offset of facility, lines 13-14) and continuously generate segments from the start node with the remaining network radius by calling the function recursiveGenerateSegs (line 15), which will be described in Algorithm 2. We do the same way to generate segments at the end node side (with the new offset is the length from *f* to end node, line 17). Each new generated segment has the weight of *f* and contains the facility identifier of *f*. This facility identifier will help the merging process when there is more than one segment of *f* generated in one edge. These new generated segments are inserted into the seg-file with the edge that contains them. In our algorithm, we use a list in order to contain edges processed completely in generating process of a facility (finished-edge-list). The edges in this finished-edge-list will not be processed during the invocation of the function recursiveGenerateSegs. After generating the segments of *f* finishes, we need to clear the finished-edge-list to start generating the segments of a new facility (line 27).

After finishing generation of the segments from a facility *f* to start node (and the end node) in Algorithm 1, if the network radius *r* is greater than the distance between *f* and the start node (and the end node), the generating process of the segments is continued from this start node (end node) with the new shortened network radius (lines 15 and 25). This process is described in Algorithm 2, which helps segments spread out the network range *f*(*r*).

In Algorithm 2, we generate all edges of the current node (i.e., the node we start generating segments). These edges are created from the neighbor list of current node, except the old node, which has been already processed (line 1). To process an edge, we need to consider two situations. In the first situation, this edge does not exist in finished-edge-list (line 5). If the length of this edge (e.g., 〈curN, neighN〉) is greater than or equals the new network radius, we only need to create a new segment between the current node and the neighbor node with its length being equal to the new radius. Then, we insert this segment into seg-file (lines 6–8). If the length of the edge is smaller than the new network radius, we create a new segment between the current node and the neighbor node, and insert this new segment into seg-file, after which we continuously generate segments from the neighbor node with the new shorten network radius (line 13). In the second situation, this edge existed in the finished-edge-list (lines 15–19). If the length of the edge is smaller than the new network radius, we only need to generate segments from the neighbor node with the new shortened network radius (line 17). This process continues until the generated segments cover the network range *f*(*r*) of the original facility.

**Figure 7:**
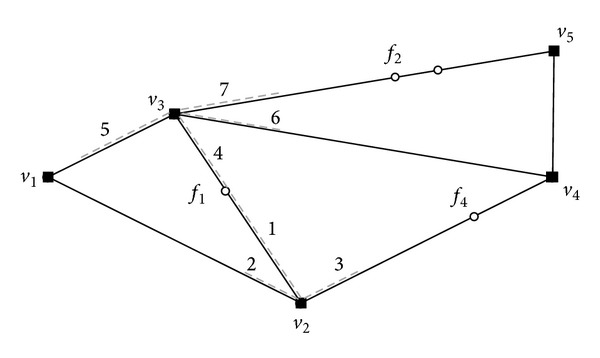
Generating segments of a facility.

Figure 7 shows the process of generating segments of facility *f*_1_ in road network shown in Figure 3. In this example, the network radius is 1.5. First of all, we generates the first segment 〈*f*_1_, *v*_2_〉 with length 1 and then two segments with length 0.5 on 2 edges 〈*v*_1_, *v*_2_〉 and 〈*v*_2_, *v*_4_〉. After that, we generate segment 〈*f*_1_, *v*_3_〉 with length 0.803 and 3 segments on 3 edges〈*v*_1_, *v*_3_〉, 〈*v*_3_, *v*_4_〉, and 〈*v*_3_, *v*_5_〉 with the same length 0.697. The numbers nearby segments show the generating order of these segments.

#### 3.3.3. Inserting Segments into Seg-File

Segments generated at step 1 are inserted into seg-file (together with containing edge information).  Algorithm 3 describes this insertion process. One important point of seg-file is that all segments on the same edge will be grouped into one record (edge-record). So, each edge-record in seg-file has the format of the form 〈edge, (segment  1, segment  2,…)〉. This seg-file is indexed by B^+^-tree. This structure of seg-file helps to find max-segments effectively.

**Figure 8:**
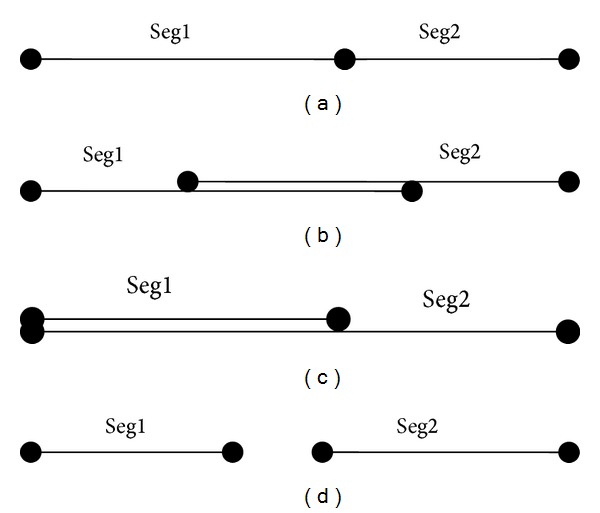
Two segments of a facility in one edge.

When we insert a segment into seg-file, if there is no edge-record of that segment in seg-file, we create a new edge-record of that segment and insert it into seg-file (lines 3-4). In case an edge-record of that segment has already existed in seg-file, we need to check if there exist any segments of the same facility in this edge-record. If this is the case, we need to merge these existing segments with the new segment (lines 7–13). Then, the mergeSegment function merges two segments into the same edge (line 8).  Figure 8 shows some situations of position of two segments in an edge. In the first three situations, the mergeSegment function returns one new segment, whereas in the last situation, it returns null (two segments cannot be merged). After updating segment list of edge-record, we update this edge-record in the seg-file (lines 15-16).

**Figure 9:**
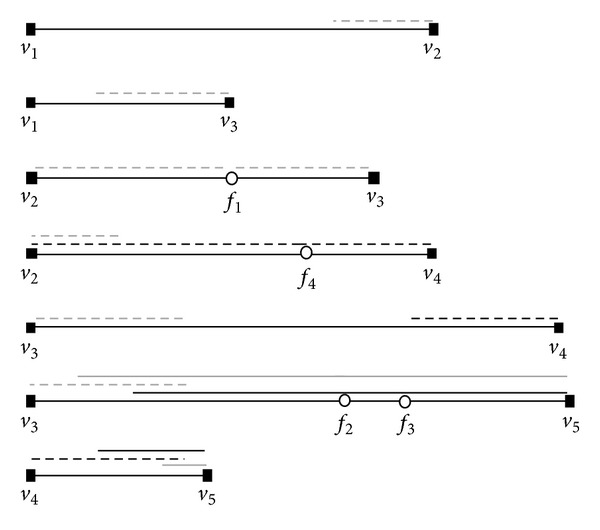
Records in seg-file.

Figure 9 shows the records in seg-file after finishing the generating segments step and inserting segments step. In the figure, the segments generated from the facility *f*_1_ are gray dotted segments, the segments generated from the facility *f*_2_ are gray solid segments, the segments generated from the facility *f*_3_ are black solid segments, and finally the segments generated black dotted segments originate from the facility *f*_4_. Each record associates with one edge (e.g., the thin solid line 〈*v*_*i*_, *v*_*j*_〉). In Figure 9, the first record associates with the edge 〈*v*_1_, *v*_2_〉 and contains one segment generated from facility *f*_1_.

#### 3.3.4. Finding Max-Segments

After finishing construction of the seg-file, Algorithm 4 is invoked, which is the process of finding max-segments from the seg-file.

In this algorithm, we find the local optimal segments in each edge-record first (line 4), after which we compare the maximum weight of segments on these edge-records, and the segments that have maximum weight are added into the list as final result (lines 6–14). The process of finding local optimal segments is processed by function lineSweep, which is the line version of algorithm* plane Sweep* proposed in [11].

**Figure 10:**
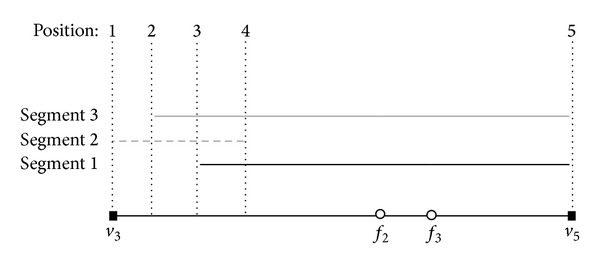
An example of* line Sweep* algorithm.

Figure 10 illustrates the algorithm* line Sweep* on the record associated with the edge 〈*v*_3_, *v*_5_〉. Assuming that we are sweeping on an edge (e.g., 〈*v*_3_, *v*_5_〉), if we meet a start node of a segment (e.g., positions 1 in the case of segment 2,…) the weight of this segment will be included in the calculation of local maximum weighted segment; in case we meet an end node (e.g., position 4 in the case of segment 2,…), we will remove the weight of this segment from the calculation. In the figure, the segment from position 3 to position 4 on edge 〈*v*_3_, *v*_5_〉 is the local maximum weighted segment of this record.

**Figure 11:**
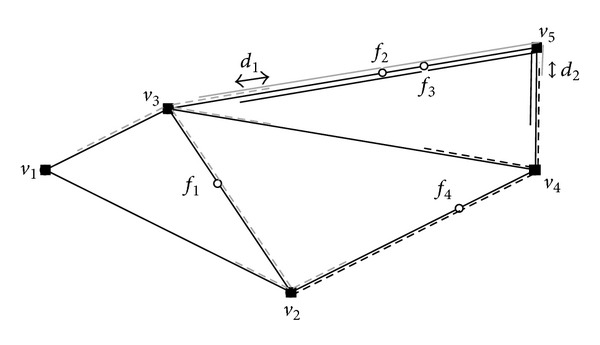
Max-segments in road network.

After finishing the finding max-segments step, from Figure 11, we can see that two segments  *d*_1_ (in edge 〈*v*_3_, *v*_5_〉) and  *d*_2_ (in edge 〈*v*_4_, *v*_5_〉) are max-segments with maximum weight (e.g., 3) in the example of Figure 3 (we assume that the weight of each facility is 1).

## 4. Performance Evaluation

### 4.1. Simulation Setup

**Figure 12:**
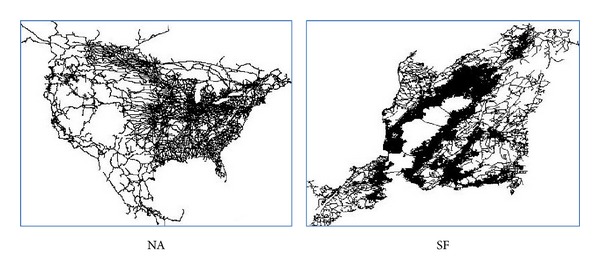
Two datasets used in experiments.

We use two real datasets, namely, North America (NA) road network and San Francisco (SF) road network. These datasets are depicted in Figure 12. The NA dataset is obtained from http://www.cs.fsu.edu/~lifeifei/SpatialDataset.htm and the SF dataset is obtained from [15]. The cardinalities of datasets are shown in Table 1.

Because this is the first work for processing MaxRS queries in a road network database, we develop a naive algorithm to compare with our proposed algorithm. The naive algorithm uses an unstructured seg-file, and thus the generated segments are inserted directly to seg-file in step 2 (segments on the same edge are not grouped into one edge-record). In step 3, the naive algorithm reads the segments from seg-file, groups segments in the same edge, and finds max-segments.

We use disk-based storage model to store very large road network databases, so in our simulation, the performance metric is the number of I/O's, which is the number of read/write blocks from files. We do not consider CPU time because it is dominated by I/O cost [10, 12, 16]. The default values of the parameters are shown in Table 2.

### 4.2. Simulation Results

#### 4.2.1. Effect of the Number of Facilities

**Figure 13:**
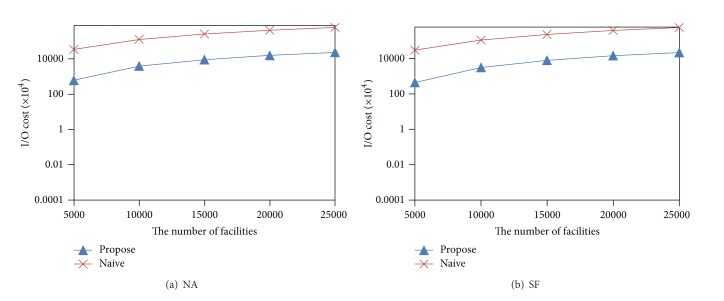
Effect of the number of facilities.

Figure 13 shows the effect of the number of facilities on the I/O cost. For both datasets NA and SF, when the number of facilities increases, the I/O cost increases. However, the proposed method is much less sensitive to this parameter than the naive algorithm.

#### 4.2.2. Effect of the Network Radius

**Figure 14:**
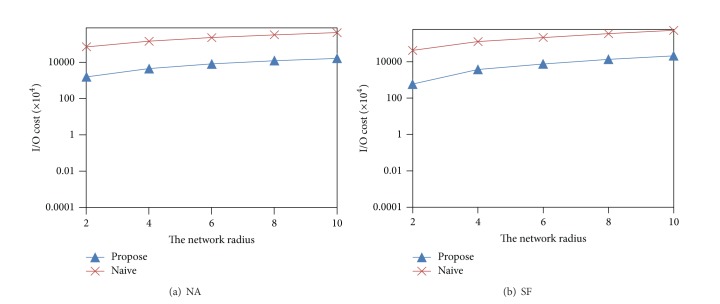
Effect of the network radius.

Figure 14 shows the results for the varying of network radius (network range). When the network radius increases, the number of segments increases, and thus the I/O cost also increases. The increment of I/O cost in SF dataset is greater than NA dataset because we can see the destiny of edges in SF is higher than NA. Therefore, the number of generated segments of SF is more than NA.

#### 4.2.3. Effect of the Buffer Size

**Figure 15:**
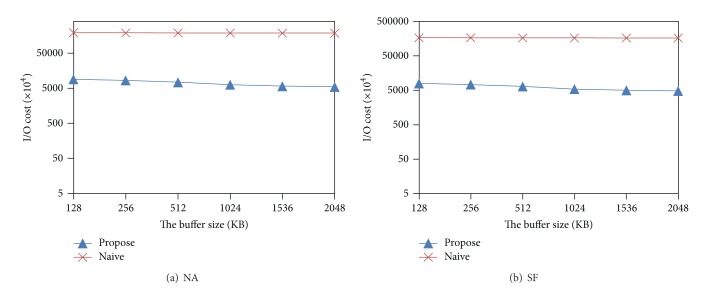
Effect of the buffer size.

Figure 15 shows the results for the varying of buffer size. Although both algorithms have better performance as the buffer size increases, the proposed algorithm is more sensitive to the size of buffer than the naive algorithm.

#### 4.2.4. Effect of the Block Size

**Figure 16:**
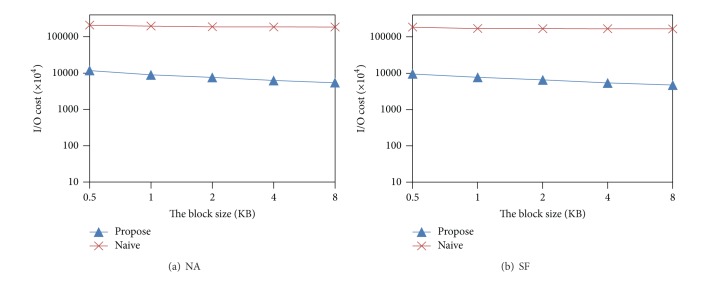
Effect of the block size.

Figure 16 shows the results for the varying of block size. We can see that when the block size increases, the I/O cost decreases. This is because as the block size increases, the number of objects stored in a block also increases, which causes the number of read/write blocks to decrease. Similar to the buffer size case, the proposed algorithm is more sensitive to the size of block than the naive algorithm.

## 5. Related Work

In this section, we review related work on facility optimization location problem in general and MaxRS problem in particular.

*Facility Optimization Location Problem*. MaxRS problem can be seen as an instance of facility location optimization problem, which has been studied extensively in current years. The aim of this facility location optimization problem is to find an optimal location to maximize/minimize an objective function. Cabello et al. introduced and investigated optimization problems according to the bichromatic reverse nearest neighbor (BRNN) rule [17], while Wong et al. [18] studied a related problem called MaxBRNN; find an optimal region that maximizes the size of BRNNs. These two problems are studied in *L*_2_ space. Du et al. [19] proposed that the optimal-location query returns a location with maximum influence, where the influence of a location is the total weight of its RNNs. In the extension version of [19], Zhang et al. [20] proposed and solved the min-dist optimal-location query.

There are some studies, specially, about facility location optimization in road network database. Xiao et al. [21] have studied about optimal location queries in road network, with the introduction of three important types of optimal location queries: competitive location query, MinSum location query, and MinMax location query. Yan et al. also proposed some algorithms for finding optimal meeting point, which have smallest sum of network distances to all the points in a set of points *Q* in road networks [22].

*MaxRS Problem*. Imai and Asono proposed an optimal algorithm for the max-enclosing rectangle problem [11] with the time complexity being *O*(*n*log⁡⁡*n*); n is the number of rectangle. Nandy and Bhattacharya also presented another algorithm which is based on interval tree data structure with the same cost [14]. Those algorithms are internal memory algorithms. Choi et al. [10] proposed an algorithm for solving MaxRS problem in the case of external memory with optimal I/O cost. Tao et al. [12] proposed a new problem called (1 − *ε*)-approximate MaxRS which returns a solution that can be worse than optimal solution by a factor at most *ε*; *ε* is an arbitrary small constant between 0 and 1.

Another version of MaxRS problem is maximizing circular range sum (MaxCRS) problem. This is a circle version of MaxRS problem with the boundary being a circle. Chazelle and Lee [23] proposed an algorithm for solving the max-enclosing circle problem with the time complexity being *O*(*n* ^2^). As max-enclosing circle problem is 3SUM-HARD [24], in which the best algorithm takes *O*(*n* ^2^) time, many studies used approximate approaches to solve max-enclosing circle problem. Aronov and Har-Peled [25] give a Monte-Carlo (1 − *ε*)-approximation algorithm for unweighted point sets that runs in *O*(*nε* ^−^2log⁡⁡*n*) time; this algorithm can be extended to the weighted case, giving an algorithm that uses *O*(*nε* ^−2^log⁡^2^⁡*n*) time. de Berg et al. [26] proposed another approximation algorithm for max-enclosing circle problem with time complexity *O*(*n*log⁡⁡*n* + *nε* ^−3^). The MaxCRS problem is also proposed in [10] by a novel reduction that converts the MaxCRS problem to the MaxRS problem.

## 6. Conclusions

The MaxRS problem can be used in location-based applications to find the most profitable service place or the most serviceable place. All of previous studies are stated in Euclidean distance; however, in many location-based applications, the network distance is used instead of Euclidean distance. This paper proposed an efficient algorithm for solving the MaxRS problem in road network database. We proposed an external-memory algorithm, which is suitable for large dataset of road network. In our algorithm, all optimal locations (max-segments) on the network will be returned while all previous methods only return one result. This can help clients of diverse interests choose their own best locations by considering other additional conditions. For the future works, we plan to improve our method and calculate the complexity of algorithm.

## Figures and Tables

**Algorithm 1:**
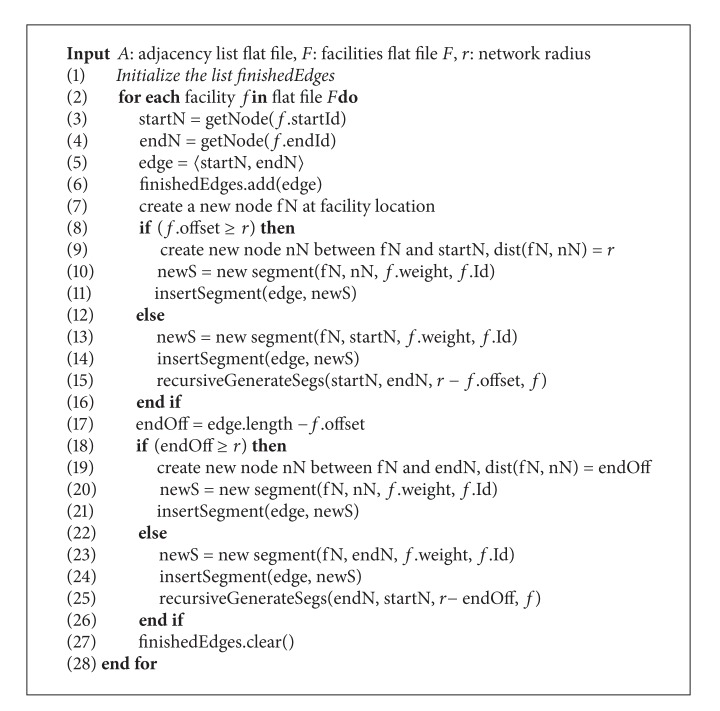
generateSegments(*A*, *F*, *r*).

**Algorithm 2:**
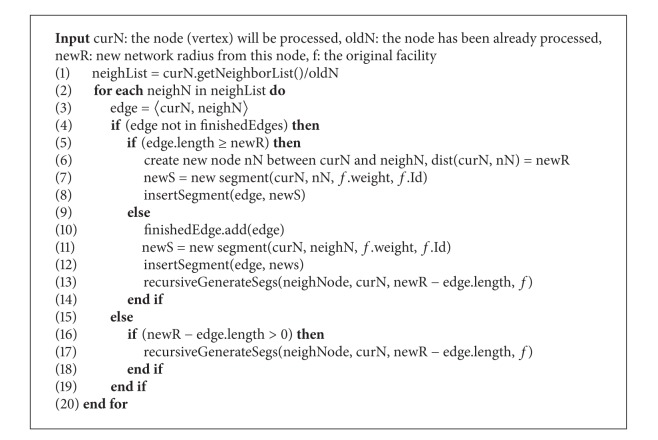
recursiveGenerateSegs(curN, oldN, newR, *f*).

**Algorithm 3:**
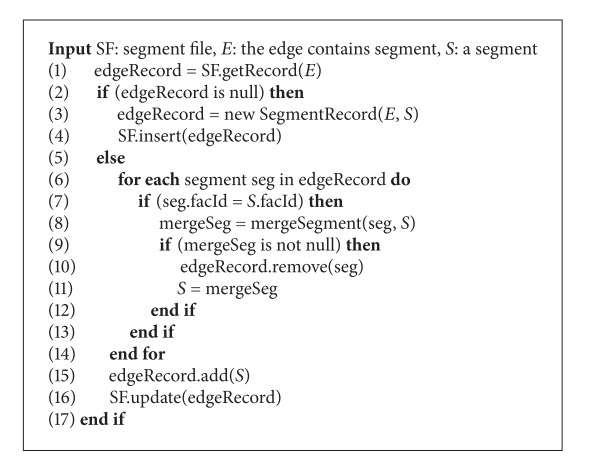
insertSegment(SF, *E*, *S*).

**Algorithm 4:**
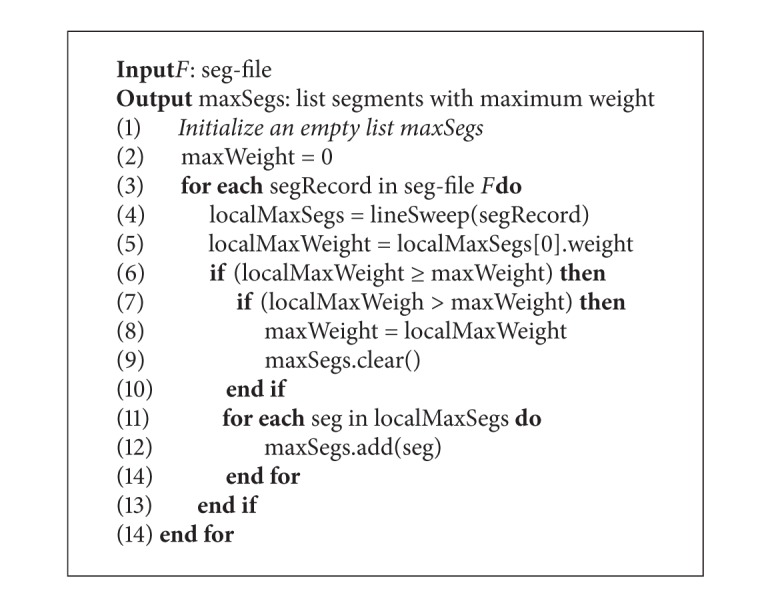
findMaxSegments(*F*).

**Table 1:** Cardinalities of real datasets.

Dataset	Nodes	Edges
NA	175813	179179
SF	174956	223001

**Table 2:** The default values of parameters.

Parameter	Default value
Facilities	12500
Block size	4 K
Buffer size	1024 K
Network radius	5
